# Industrial action by healthcare workers in Nigeria in 2013–2015: an inquiry into causes, consequences and control—a cross-sectional descriptive study

**DOI:** 10.1186/s12960-016-0142-7

**Published:** 2016-07-27

**Authors:** Obinna Ositadimma Oleribe, Iheaka Paul Ezieme, Olabisi Oladipo, Ezinne Patience Akinola, Deborah Udofia, Simon D. Taylor-Robinson

**Affiliations:** 1 Excellence & Friends Management Care Centre (EFMC), Abuja, Nigeria; 2 Hepatology Unit, Imperial College London, 10th Floor, QEQM Building, St Mary’s Hospital Campus, South Wharf Road, W2 1NY London, United Kingdom; 3 No 8, Excellence and Friends Street, Dutse, P. O. Box 200, PSIN, 901101 Abuja, Nigeria

**Keywords:** Nigeria, Healthcare worker, Strikes, Industrial actions, Health system

## Abstract

**Background:**

Nigeria has suffered from several healthcare workers’ strikes in the past 36 months, involving all categories of health workers. Frequent healthcare workers’ strikes result in the closure of public healthcare institutions preventing Nigerians’ access to quality health services. The purpose of this study was to identify the root cause(s) of strikes by healthcare workers, their effects on the health system and possible solutions to prevent, or at least reduce, industrial action.

**Methods:**

A cross-sectional descriptive survey was used to execute this study between February and March 2015. A self-administered questionnaire with both closed- and open-ended questions was used for this study. Data were analysed using EpiData™ and SPSS 21. Simple frequencies and chi-square analysis were carried out.

**Results:**

A total of 150 healthcare workers participated in the study. Sixty-two (41.3 %) participants were males, 86 (57.3 %) married, 90 (60.0 %) Christians and 119 (79.3 %) graduates, and about half of the participants earn less than N129 000.00 (US$ 737.00) per month. Less than half of the participants (43.6 %) supported industrial actions. Poor healthcare leadership and management were cited as the most common (92 %), as well as the most important (43.3 %), cause of healthcare worker strikes in Nigeria. Other causes cited were a demand for higher salaries and wages (82 %), infrastructural issues (63.3 %) and inter-personal issues (61.3 %). Only 2.0 % rated current healthcare management as excellent, while 24.0 % rated it as very good. Several strategies were cited towards improving healthcare management.

**Conclusions:**

The findings of this study differ from previous studies that identified demand for increased salaries and wages as the most common cause of healthcare workers’ strikes in Nigeria. Identified causes of these continued strikes, especially inadequate healthcare leadership/management, must be tackled in order to eliminate industrial action by healthcare workers. Training doctors in health management and leadership towards building skilled physician leaders is a strategy that is long overdue in Nigeria.

## Background

Workers’ strikes involve the collective withholding of labour/services by a group of workers for the purpose of extracting certain concessions or benefits and are usually intended for the economic benefits of the strikers [1]. In Nigeria, the law allows all workers to form or join unions, with the exception of members of the armed services, the police force, firefighters, Central Bank employees and customs and excise staff [2]. While the first recorded strike action in history took place during the reign of Ramses III in the twelfth century BC, health workers’ strikes have remained commonplace throughout history as well as in Nigeria [3]. The first nationwide strike by the organized workforce in Nigeria was on 21 June 1945 by about 150 000 clerical and non-clerical workers in the Nigerian civil service, demanding better wages in response to the rising cost of living brought about by the Second World War [4]. In the last 36 months, the Nigerian health system has experienced more than eight different strikes involving doctors, nurses and allied healthcare workers [5–10]. These strikes have negatively impacted on the healthcare system, leading to several avoidable deaths, complications and outgoing medical tourism, as the wealthy seek health services abroad [11]. The impact of these strikes is worst when they occur at periods of national health emergencies such as the recent Ebola viral disease outbreak, Lassa fever or cholera outbreaks or even man-made emergencies like Boko Haram suicide bombings with mass casualties [12]. Reasons abound why healthcare workers go on strike in true underlying causes of industrial action Nigeria, and these include career stagnation, perceived discriminatory policies and demoralization from working in systems with poor infrastructure, manpower shortages and poor personal remuneration [3]. However, in recent times, there has been a division of opinion on pinpointing the true underlying causes of industrial action [1, 13].

As health workers’ strikes continue to be seen and accepted as normal behaviour in the Nigerian heath sector, we decided to study the characteristics of health workers’ industrial action in Nigeria. The purpose of this study was to identify the root cause(s) of healthcare worker strikes and their effects on the health system and proffer possible solutions aimed at preventing, or at least reducing, industrial actions. This study also aimed at stimulating discussions amongst health workers and managers on the best grievance management strategies for the healthcare industry and hospital leadership with sustainable improvement in health outcomes.

## Methods

A cross-sectional descriptive survey approach was used to execute this study between February and March 2015. A questionnaire with both closed- and open-ended questions was developed; pre-tested for accuracy, analyzability, acceptability and ambiguity; and used for this study. The study population was selected from the healthcare worker population in Abuja, the Nigerian Federal Capital Territory, which is home to almost 200 government-owned healthcare facilities offering primary, secondary and tertiary healthcare. Six government-owned healthcare facilities (four secondary healthcare facilities, one comprehensive healthcare centre and one primary healthcare centre) were selected by convenience sampling. One hundred and fifty health workers who were met at their duty posts during lunch hour within the premises of their respective hospitals were enlisted for the study. Healthcare workers were adequately informed of the study, and those who agreed to participate were enlisted into the study. Verbal consent was received from all participants prior to the administration of the questionnaires. The questionnaires comprised of 40 questions and were self-administered. Study variables include causes of strikes, their implications and suggested solutions. The completed questionnaires were retrieved from each individual on an agreed date and time and screened for accuracy and completeness.

Data were entered into EpiData™ [14] from where information was exported into SPSS 21 [15] for analysis after data cleaning and validation. Simple frequencies and chi-squares were performed and relevant tables developed. Age, educational qualification, marital status and number of years in service were re-coded for the chi-square analysis. Recoding of variables saw all participants grouped into graduates and non-graduates, married and single (with widows classified as singles), below 5 years in service and 5 years and above and workers below 40 years of age and 40 years of age.

## Results

A total of 150 questionnaires were printed and distributed to healthcare workers, and all were retrieved within the study period, giving a response rate of 100 %. The 100 % response rate resulted from the use of a questionnaire with simple questions, which did not take more than 10 min to fill. An added advantage was that the investigators had an established relationship with the hospitals where the healthcare workers worked. Of the 150 healthcare workers enlisted for the study, 62 (41.3 %) were males. Participants had an age range of 26 to 52 years and average age of 33.9 years (S.D. = 5.36). Moreover, 122 (83 %) of the 147 indicated their age were less than 40 years old. Of the 150 participants, 86 (57.3 %) were married, 90 (60.0 %) were Christians and 119 (79.3 %) were graduates with either a BSc (106, 70.7 %) or MSc (13, 8.7 %). Income-wise, half of the participants earn less than N129 000.00 (US$ 737.00) per month. Table 1 shows the socio-demographic characteristics of the study participants.

**Table 1:** Socio-demographic characteristics of study participants

SN		Number	Percent
Gender of participants			
	Gender		
1	Male	62	41.3
2	Female	88	58.7
	*Total*	*150*	*100*
Age of respondents			
	Ages		
1	25–29	26	17.7
2	30–34	68	46.3
3	35–39	28	19.0
4	≥40	25	17.0
	*Total*	*147*	*100*
Marital status of participants			
	Marital status		
1	Married	86	57.3
2	Single	61	40.7
3	Widow	3	2.0
	*Total*	*150*	*100*
Religion of practice of participants			
	Religion		
1	None	4	2.7
2	Christianity	90	60.8
3	Islam	54	36.5
	*Total*	*148*	*100*
Monthly income of participants			
	Monthly income		
1	10 000–49 000	21	14.19
2	50 000–89 000	21	14.19
3	90 000–129 000	33	22.30
4	130 000–169 000	26	17.57
5	170 000–199 000	22	14.86
6	>199 000	25	16.89
	*Total*	*148*	*100*
Academic qualification of respondents			
	Highest academic qualification		
1	First school leaving certificate	1	0.7
2	SSCE/WAEC	2	1.3
3	Certificates/diploma	28	18.7
4	Bachelors	106	70.7
5	Masters and above	13	8.7
	*Total*	*150*	*100*

More than half of the respondents (85, 56.7 %) had worked in the health system for less than 5 years with 70 % (105) in public health service. Physicians constituted 20.7 % of the respondents with nurses and pharmacists constituting 17.3 % each (Table 2). While 76.67 % of the respondents believed that the health system in Nigeria is understaffed, 73.3 % were satisfied with both their current earnings and their working conditions. However, 46 % (69) were facing challenges of human resource shortages in their places of work, poor infrastructure (36 %) and lack of financial incentives to work (22.7 %).

**Table 2:** Occupational history of participants

SN		Number		Percent
Number of years in public health system				
	Years in service			
1	<5	85		56.67
2	5–9	40		26.67
3	10–14	14		9.33
4	15–19	9		6.00
5	20–24	1		0.67
6	>24	1		0.67
	*Total*	*150*		*100.01*
Current place of employment of respondents				
	Current place of employment			
1	Public service		105	70.0
2	Private		45	30.0
	*Total*		*150*	*100*
Current title in place of employment				
	Title of respondents			
1	Doctors		31	20.7
2	Nurses		26	17.3
3	Pharmacist		26	17.3
4	Lab scientist		23	15.3
5	Admin officer		20	13.3
6	Community ext. worker		17	11.3
7	Physiotherapist		4	2.7
8	Radiographer		2	1.3
9	Optician		1	0.7
	*Total*		*150*	*100*
Respondent who think Nigeria healthcare system is understaffed				
	Nigeria understaffed			
1	Yes		115	76.67
2	No		10	6.67
3	Undecided		25	16.67
	*Total*		*150*	*100.01*
Satisfaction with current earning				
	Satisfied			
	Yes		110	73.3
	Undecided		21	14.0
	No		19	12.7
	*Total*		*150*	*100*
Satisfaction with working conditions				
	Satisfied			
1	Yes		110	73.3
2	No		19	12.7
3	Undecided		21	14
	*Total*		*150*	*100*
Problems/difficulties faced in respondents’ places of work				
	Problems			
1	Manpower shortage		69	46.0
2	Poor infrastructure		54	36.0
3	Lack of incentives		34	22.7
4	Stagnation		16	10.7
5	Lack of professional development		13	8.7

When asked about their attitude to health worker strikes, 43.6 % were in support of the industrial actions (Table 3).

**Table 3:** Health workers attitude to strikes in Nigeria

SN		Number	Percent
Support of strike by health workers			
	Support for strike		
1	Yes	65	43.6
2	No	56	37.6
3	Undecided	28	18.8
	*Total*	*149*	*100*
Suggested common causes of strikes in Nigeria (participants free to name more than one)			
	Common causes of strike		
1	Leadership/management	138	92.0
2	Demand for higher salaries and wages	123	82.0
3	Infrastructure	95	63.3
4	Inter-personal issue	92	61.3
5	Funding	68	45.3
6	Inadequate workers	67	44.7
7	Inadequate tools	44	29.3
8	Inadequate training	13	8.7
9	Disagreement on some issues	12	8.0
10	Politics	6	4.0
11	Patient load	2	1.3
12	Mass sack	2	1.3
13	Others	1	0.7
Most common causes of strikes in Nigeria			
	Most important causes		
1	Leadership/management	65	43.3
2	Inter-professional issues	33	22.0
3	Demand for higher salaries/wages	26	17.3
4	Funding	17	11.3
5	Inadequate tools	1	0.7
6	Disagreement on some issues	1	0.7
7	Infrastructure	1	0.7
8	No response	6	4.0
	*Total*	*150*	*100.0*
Common implication of strikes in the health sector			
	Implications		
1	Disruption of healthcare	145	96.7
2	High referral to private hospitals	99	66.0
3	Patients loss to follow-up	84	56.0
4	High private hospital cost	26	17.3
5	Mismanagement by quacks	26	17.3
Suggested solution to recurrent strikes in health industry			
	Suggested solutions		
1	Strengthen health system	124	82.7
2	Motivate health workers financially and professionally	74	50.7
3	Carry workers along in decision making	65	43.3

All were concerned with the effects of the strike on the patients (150, 100 %)

### Causes of strikes

Healthcare leadership and management issues were cited as the most common (92 %), as well as the most important (43.3 %), causes of health workers’ strikes in Nigeria. Other common causes were demand for higher salaries and wages (82 %), infrastructural issues (63.3 %) and inter-personal issues (61.3 %).

On the scale of importance, inter-professional issues and demand for higher salaries and wages were cited as the second and third most important causes of healthcare workers’ industrial action in Nigeria (Table 3).

### Implications of strikes

Respondents also cited disruption of patient care (96.7 %) as the most common implication of health worker strikes in Nigeria. Perceived consequences and reasons for further discontent were high referral rates to private hospitals (66.0 %), patient loss to follow-up (56.0 %), mismanagement by alternative healers and high private hospital costs (17.3 %). Other common effects of strikes by healthcare workers on patients and healthcare systems cited by the respondents were an increase in financial burden on patients; increased morbidity and mortality, especially amongst the poor; collapse of publically funded health facilities; loss of confidence in the health system; unequal access to quality medical care; emigration of qualified health workers; increased spread of contagious diseases; and negative impact on national productivity.

### Suggested solutions

Although all respondents were concerned with the effects of the strikes on patients, they were of the opinion that strengthening of the healthcare system (82.7 %), improving financial and professional motivation of health workers (50.7 %) and involving healthcare workers in decision-making (43.3 %) were possible solutions to the plethora of strike action in Nigeria (Table 3). On specific changes in the health industry that would bring about an end to the numerous strikes in Nigeria, the respondents cited leadership and management (88.7 %), salaries of workers (68.0 %), financial management (61.3 %) and infrastructural changes (48.0 %) as crucial. However, when asked to state the most important change each of them would like to see, a change in leadership and management (64.7 %) was said to be the most important factor. Others were improved financial management (13.3 %) and enhanced salary of workers (11.3 %) as shown in Table 4. The respondents were of the view that the following would eliminate or prevent the occurrence of health workers’ strikes in Nigeria: (1) government sensitivity to the needs and demands of healthcare workers; (2) negotiations with all parties involved; (3) the government taking the agitations of the healthcare sector workers more seriously; (4) salary increase to be effected; (5) changes to labour laws; (6) reduction of corruption and increase in transparency; (7) proper outlining of job descriptions for various healthcare providers; (8) training and re-training of staff to understand the value of efficiency; (9) good inter-professional relationship; and (10) creation of policies that improve the state of health institutions.

**Table 4:** Participants suggested changes or solutions to health workers strikes in Nigeria

SN		Number	Percent
Changes they would want to see			
	Changes		
1	Leadership/management	133	88.7
2	Salaries of workers	102	68.0
3	Financial management	92	61.3
4	Infrastructure	72	48.0
5	Work packages	17	11.3
6	Promotion guidelines	12	8.0
7	Guidelines	7	4.7
8	Ownership of facility	2	1.3
Most important changes they will like to see			
	Most important changes		
1	Leadership/management	97	64.7
2	Financial management	20	13.3
3	Salary of workers	17	11.3
4	Infrastructure	7	4.7
5	Work place	1	0.7
6	Guidelines	1	0.7
7	Available and affordable system for all	1	0.7

When asked to rate the current hospital leadership and management in terms of effectiveness and efficacy, only 2.0 % rated it as excellent, while 24.0 % rated it as very good. The rest, as shown in Table 5, rated it as just good or average. To improve health system leadership, the respondents suggested periodic evaluation (86.0 %), more training (84.0 %) and “in-service” management/leadership training (82.0 %) for healthcare workers. Only 9.3 % (14) cited pre-service courses as improvement strategies for healthcare leadership. However, 26 % asked that leadership of the health system be open to all healthcare workers. The study went on to ask the participants, “who should lead the health team?” To this question, 54.67 % were of the view that doctors should lead the team, while the rest suggested that any professional (26.67 %) or health administrator (18.67 %) could head the medical team (Table 5).

**Table 5:** Leadership of the health institutions, rating and suggested ways of improvement

SN		Number	Percent
Rating of hospital leadership/management			
	Rating		
1	Excellent	3	2.0
2	Very good	36	24.0
3	Good	101	67.3
4	Average	10	6.7
	*Total*	*150*	*100*
How can health leadership be improved			
	Improvement method		
1	Periodic evaluation	129	86.0
2	More training	126	84.0
3	In-service management/leadership training	123	82.0
4	Open to all health workers	39	26.0
5	Pre-service courses	14	9.3
Most Important of all the improvement methods			
	Most important improvement method		
1	Periodic evaluation	74	49.3
2	More trainings	32	21.3
3	In-service management/leadership training	28	18.7
4	Open to all health workers	8	5.3
5	No comments	8	5.3
Who should be the head of the hospital			
	Who should be the head		
	Doctors	82	54.67
	Any professional	40	26.67
	Health administrator	28	18.67
	*Total*	*150*	*100*

As this study took place during an ongoing health worker strike, the opinions of the healthcare workers were sought on the current strike. While 78.5 % (117) were aware of the reason behind the current strike in the country, only 45.0 % (67) agreed to the strike and 45.3 % (68) were of the view that the strike would achieve the desired outcomes (Table 6).

**Table 6:** Current strike, knowledge, attitude and practices

SN		Number	Percent
Knowledge for the reason for present strike			
	Knowledge of the reason for the present strike		
	Yes	117	78.5
	No	20	13.4
	I do not know	12	8.1
	*Total*	*149*	*100*
Agreement with the reason for the strike			
	Agreed		
1	Yes	67	45.0
2	No	54	36.2
3	Undecided	28	18.8
	*Total*	*149*	*100*
Do you think the strike will yield desired outcome?			
	Will yield desired outcome		
	Yes	68	45.3
	No	6	4.0
	Uncertain	76	50.7
	*Total*	*150*	*100*
Are union activist beneficial to the overall objective?			
	Union activist beneficial		
	Yes	143	95.3
	No	4	2.7
	Undecided	3	2.0
	*Total*	*150*	*100*
Can you boycott the strike for the sake of patients?			
	Can boycott strike		
	Yes	69	46.3
	Undecided	52	34.9
	No	28	18.8
	*Total*	*150*	*100*
Collective bargaining useful in dispute			
	Collective bargaining		
	Yes	150	100
	Undecided	0	0
	No	0	0
	*Total*	*150*	*100*

All agreed on a team approach to healthcare delivery (150, 100 %)

To mitigate the impact of strikes, the respondents had advice for the management and leadership of healthcare institutions and to patients and their relations. To the healthcare workers, it was suggested that they work corporately as a team, understand their patients, put more effort in their work and pursue further postgraduate studies to improve their clinical practice, while working to understand the hierarchy in the healthcare system, improving relationships between medical and non-medical staff, but making the health of the patients paramount. To the healthcare leadership, it was suggested that conducive working environments be created for staff, in addition to being dedicated to duty, willing to listen to the grievances of healthcare workers, ensuring that due process is followed in relation to promotion and confirmation of staff, ensuring prudent management of available resources, learning to serve and to provide consensus amongst their team workers and be a little more flexible with the governmental position. To the patients, it was suggested that they create patient associations or defence coalitions and also demand more from the government and healthcare workers with respect to healthcare quality, understand the challenges of healthcare workers and join forces with healthcare workers during strikes to get the government to listen and not to turn against healthcare workers.

To improve hospital management and working conditions, the respondents were of the view that the government should employ more workers, provide better infrastructure, invest more on healthcare, organize seminars and training, monitor the promotion process and root out corruption, while improving staff welfare packages and better equipping the health centres.

All chi-square analyses of various variables were not significant (Table 7).

**Table 7:** Bivariate analysis for gender, age and education as against support for strike and hospital leadership rating as dependent variable

		Gender		Total
		Male	Female	
Support for strike	Yes	25	40	65
	No	37	47	84
	Total	62	87	149
*X* ^2^ 0.471, *P* value 0.493				
Hospital leadership rating	Performing to standard	13	26	39
	Not performing to standard	49	62	111
	Total	62	88	150
*X* ^2^ 1.391, *P* value 0.238				
		Age		
		<40	≥40	
Support for strike	Yes	54	10	64
	No	67	15	82
	Total	121	25	146
*X* ^2^ 0.180, *P*-value 0.671				
Hospital leadership rating	Performing to standard	29	9	38
	Not performing to standard	93	16	109
	Total	122	25	147
*X* ^2^ 1.619, *P* value 0.203				
		Education		
		Graduate	Non-graduate	
Support for strike	Yes	54	11	65
	No	65	19	84
	Total	119	30	149
*X* ^2^ 0.739, *P* value 0.390				
Hospital leadership rating	Performing to standard	33	6	39
	Not performing to standard	86	25	111
	Total	119	31	150
*X* ^2^ 0.897, *P* value 0.344				

## Discussion

Strikes amongst healthcare workers are not rare events in most countries, as is the case in Nigeria. In recent years, there has been an increasing number of healthcare worker strikes across the nation, some national, others regional or state-based [3, 5–10]. It is not surprising to discover that the primary cause of most national healthcare worker strikes in Nigeria is demand for higher salaries and wages. For instance, of the 24-point reason for the 2014 National Medical Association (NMA) doctors’ strike, only one made reference to health trust funds to enhance the upgrading of hospitals in Nigeria [16]. The rest focused on doctors’ welfare, salaries and wages, career enhancement and other welfare issues. A vital finding from the present study, however, is that poor healthcare leadership and management is deemed to be a more important cause of discontent than personal welfare issues as the root cause of Nigerian healthcare worker strikes (Table 3).

Healthcare workers’ strikes also exist in other countries, echoing what was seen in the United States in the 1960s and 1970s, involving the active participation of all categories of healthcare workers and in all kinds of healthcare institutions. In nations where unionism is not compulsory, such as Canada, strikes by healthcare workers are linked to economical, rational, ideological commitment, professional disaffection or social malleability [17]. This is not seen in Nigeria as all healthcare workers, by legislation, are members of one union or the other—sometimes against their will. As suggested by Adebimpe and colleagues, the demand for better salary and welfare was the most common cause of strikes in Lagos, Nigeria, followed by disagreement on a variety of work-related principles [11].

### Impact of strikes on patients

The consequences of strikes on the healthcare system are enormous. Consequences of healthcare workers’ strikes in the United States in the 1970s, which Wolfe documented in an editorial in the American Journal of Public Health in 1979, included revenue losses to the hospital and increased death on transit, as patients are transferred from one centre to the next [1]. Adebimpe and colleagues in Lagos, Nigeria, had similar findings. In Lagos, they discovered that participants believed that health workers’ strikes led to disruption of healthcare services, discharge of patients from hospital without completeness of care and limited care to clients and led to high rate of referrals to private hospitals [11]. In the same vein, Ogunbanjo and Knapp van Bogaert identified two classes of consequences—on the patients and on the healthcare workers [3]. For patients, work loss (if employed), wasted money for transport, treatment delays, prolongation of suffering, irreversible damage to health, dangerous drug interruptions and death were the documented consequences of strikes, while financial gain and improved working conditions which may contribute to less emotional pressure and even a degree of dissuasion from emigrating may be gains of strikes to healthcare workers [3]. This shows that the consequences of healthcare worker strikes are greatly skewed in favour of healthcare workers. However, more work is needed on the economic, societal and political consequences of strikes in Nigeria.

The authors believe that the freedom to choose to be or not to be a member of a union and the freedom to decide to join or not to join a strike action are part of human rights and should be enshrined in the Nigeria Health Act and respected. This may be a useful mitigation against further compulsory strike action. We believe that modalities for the calling for strikes should be reviewed and all workers given equal opportunities to participate in this critical decision (with one man, one vote) so that it does not serve only the purposes of the few in leadership of the unions. We recommend that a general assembly or meeting should be called in which all workers are allowed to vote privately for or against the strikes before strikes are called. Laws should be put in place to support strikes only when more than 60 % of the workers are in favour of the strike action. This is already in practice in many countries of the world and should be replicated in Nigeria [18]. We also believe that strengthening of the health system and appropriate reward systems for healthcare workers require good health management and leadership skills to achieve. To translate this into reality, educating the new and emerging key “physician leaders” should be a fundamental step towards a reformed and revitalized health industry in Nigeria. Although presently, many people are of the view that doctors should continue to lead and head hospitals, we are of the opinion that this monopoly of doctors on healthcare leadership should be reviewed, and only those with prerequisite leadership skills training be appointed to leadership positions. This is without any prejudice to their leadership role in all technical matters concerning patient care. Appointment to managerial/leadership positions should not just be based on number of years in practice nor on technical qualifications but on leadership and management expertise and qualifications.

### Recommendations

It is true that conflicts and confrontations are inevitable in the health industry, but are health workers’ strikes the way out? What are some of the ethical and conceptual issues [1]? The International Labour Office, a United Nations agency, refers to essential services as a class of occupations that have been legislated by a government to have special restrictions with respect to labour actions, such as not being allowed to legally strike. Top in this class is the healthcare/hospital sector [19]. To curtail the effects of physicians’ strikes in Nigeria, the Medical and Dental Council of Nigeria (MDCN) on 23 June 2011 banned doctors’ strikes, because, according to the regulatory body, they cited that it is unethical for doctors to go on strike, as strikes are against the Hippocratic Oath that makes the health of the patient every doctor’s first consideration, given that doctors have vowed to always maintain the utmost respect for human life [20, 21]. However, with the numerous strikes since then by doctors, it shows that they are not willing to respect this guideline. What is more of a concern is MDCN’s inability to sanction any doctor despite their published stand on it (except in extreme conditions and with MDCN permission). The body is backed by law but lacks the will to follow through execution of punitive measures on healthcare workers who break the law. Even if the doctors respect this ban, will this eradicate the causes of strikes we identified in this study? The underlying issue is the faulty healthcare system in existence in Nigeria, ranging from ineffective leadership in the healthcare industry, poor working conditions of workers and inadequate infrastructure and equipment. The MDCN cannot sanction doctors who embark on strikes, although the regulatory body is aware of the prevailing poor working conditions. In spite of this, it is critical that healthcare professionals, who are indispensable to the protection of the healthcare and life of patients and the public, not be allowed to withdraw their services from the sick, as abandonment of sick patients should be both an illegal and potentially criminal offence in a just society [1]. To this end, although health workers can (and may) withdraw their services from government, they cannot (and should not) withdraw their services to patients. Other countries have developed systems whereby basic services are not disrupted during strikes but are still being provided by emergency teams, so as to preserve both the right to strike and the patients’ well-being. This is being practised in Nigeria to a certain extent as emergency services are still being rendered in certain instances during strikes. However, this needs to be expanded as well. It is also critical that the issues raised by this study, particularly health system leadership, be tackled and resolved as part of the twenty-first century healthcare system reforms and healthcare system strengthening. All existing and emerging conflicts should therefore be controlled through concession and compromises from all sides of the divide, while building functional and effective health leadership. As part of solutions proffered for building capacity for health management and minimizing crisis in the health sector, literature shows that the Ethiopian Ministry of Health 2-year Masters in Health Administration (MHA) course is a useful model which could help build skilled healthcare leaders and managers working towards building the kind of healthcare system that the people want [22, 23]. Nigeria should also adapt the competency frameworks, which have worked in other nations of the world, to develop functional, effective and sustainable healthcare management courses for Nigerian health leaders [24].

The study by Botero et al. stated that richer countries regulate labour less than poorer countries do, although they have more generous social security systems [13]. Is it time, therefore, for Nigeria to deregulate unionism and make it optional to workers, especially in the health industry? Will provision of universal health coverage through a functional national health insurance scheme minimize the frequency of health workers’ strikes in Nigeria? There is need for further studies to answer these questions.

## Conclusions

In line with Adebimpe and colleagues’ conclusion, although strike actions have more negative than positive effects, and a preventable way of dispute resolution in the healthcare system, they are still very commonly seen in Nigeria. Nigerians should work to minimize strikes while building health leadership that will lead to the development of world-class best practices in the Nigerian health industry. Training doctors in health management and leadership towards building skilled physician leaders is a strategy that is long overdue in Nigeria.

### Limitations

This study was carried out during a health workers’ strike. It is possible that this could have led to an element of bias in the responses we received. A second possible source of bias was the fact that the sample population for the study was limited to one state of Nigeria. In addition, a majority of the respondents had less than 5 years’ experience in the health system, given that the field workers carrying out the study had limited access to more senior colleagues. It is possible that there may have been a different result if older healthcare workers were included as a larger proportion of the respondents, but the perceptions gained from the younger healthcare workers are still valid, nevertheless. Finally, the sample size of 150 health workers may not have been adequate to power the study to allow for generalization of the findings to a national level. A more inclusive national study is therefore recommended for the future, perhaps with questionnaires administered through national healthcare bodies, such as the West African College of Physicians (WACP).

## Abbreviations

MDCN, Medical and Dental Council of Nigeria; MHA, Masters in Health Administration; NMA, Nigerian Medical Association; SPSS, Statistical Package for the Social Sciences; WACP, West African College of Physicians
